# Maritime transportation and people mobility in the early diffusion of COVID-19 in Croatia

**DOI:** 10.3389/fpubh.2023.1183047

**Published:** 2023-08-17

**Authors:** Corentin Cot, Dea Aksentijević, Alen Jugović, Giacomo Cacciapaglia, Gianandrea Mannarini

**Affiliations:** ^1^Laboratoire de Physique des 2 Infinis Irène Joliot Curie (UMR 9012), Centre Nationale de la Recherche Scientifique (CNRS)/IN2P3, Orsay, France; ^2^Pomorski Fakultet Sveučilišta u Rijeci/Faculty of Maritime Studies, University of Rijeka, Rijeka, Croatia; ^3^Univ Lyon, Univ Claude Bernard Lyon 1, Centre Nationale de la Recherche Scientifique (CNRS)/IN2P3, IP2I Lyon, UMR 5822, Villeurbanne, France; ^4^Fondazione CMCC—Ocean Predictions and Applications Division, Lecce, Italy

**Keywords:** epidemiology, mobility, COVID-19, diffusion, data analysis

## Abstract

**Introduction:**

The outbreak of COVID-19 in Europe began in early 2020, leading to the emergence of several waves of infection with varying timings across European countries. The largest wave of infection occurred in August-September. Croatia, known for being a hotspot of tourism in the Mediterranean region, raised concerns that it might have played a role in incubating the pandemic during the summer of 2020.

**Methods:**

To investigate this possibility, we conducted a data-driven study to examine the potential influence of passenger mobility to and within Croatia, utilizing various modes of transportation. To achieve this, we integrated observational datasets into the “epidemic Renormalization Group” modeling framework.

**Results:**

By comparing the models with epidemiological data, we found that in the case of Croatia in 2020, neither maritime nor train transportation played a prominent role in propagating the infection. Instead, our analysis highlighted the leading role of both road and airborne mobility in the transmission of the virus.

**Discussion:**

The proposed framework serves to test hypotheses concerning the causation of infectious waves, offering the capacity to rule out unrelated factors from consideration.

## 1. Introduction

With the growth of human population and its impact on the environment, our societies are becoming increasingly vulnerable to new diseases, especially from viral infections of zoonotic origin. At present, just 3% of the land ecosystems are untouched by human activities (1). Furthermore, human-induced climate change is causing relocation of species and rapid migration of humans, hence increasing cross-species viral transmission risks (2). The indirect impact caused by the thawing of the Arctic permafrost also poses the risk of releasing past viral charges (3). In addition to this, the economic globalization has increased the mobility of both goods and people across countries and continents, hence facilitating the global spread of disease carriers. All these factors contribute to the transmission of viral pathogens from animal species to humans, and their rapid diffusion within the world population. The COVID-19 pandemic (4, 5) showcased this process (6). It also dramatically showed the unpreparedness of human society to face the threat of a pandemic (7) and its inability to efficiently cope with the effects, evident in the emergence of multiple epidemiological waves (8).

Hence, it has become of paramount importance to define and introduce protocols and preparedness measures that help governments, private companies, and individual citizens to face a new viral pandemic in its early phases. In this context, human mobility plays a crucial role in determining the transmission of the pathogens, highlighting the importance of restriction measures at the beginning of a pandemic (9–13). Examples include lockdowns, mobility limitations within countries, and border closures. Travel between countries and geographical regions, in fact, may have played a crucial role in the diffusion of epidemic waves within and among continents, e.g., causing multiple waves in Europe right after the lockdowns were lifted (14, 15). This effect was not widely expected in the scientific community, as diverse scenarios for the short and long term evolution of the pandemic were on the table (16). Also, it was found that diffusion of the infection within a community was mainly driven by specific *superspreader locations* (17), where more intense social interactions occur.

The fact that people mobility plays a crucial role in the diffusion of an infectious disease is widely accepted in the scientific community, and it has led to the development of mobility models, such as GLEAM (18). In the very early stages, when COVID was not yet labeled as a pandemic, the danger for various countries presented by the new respiratory disease observed in Wuhan was first evaluated using mobility models (19). Similarly, people mobility is at the root of the early diffusion in China (9) and other countries (6, 20, 21). Remarkably, diffusion models based on people mobility have also been employed at the microscopic level, to understand the infection spread in a closed room (22–25). The role of mobility has also been recognized in regional instances and for the impact on isolated communities (26). However, it remains not clear what is the specific role played by different transportation vectors. While airplanes could explain the diffusion at long distance, among far away countries and continents, at a more local regional level many vectors may play a significant role: in particular, terrestrial transportation via cars and trains, and maritime passenger traffic. The main objective of this work is to establish a methodology to quantitatively measure the impact of different passenger vectors on the early diffusion of infections, using COVID-19 as a case study. Answering this research question can help guiding decision makers to determine the first responses to a new pandemic, hence improving the preparedness of the whole society.

**Figure 1:**
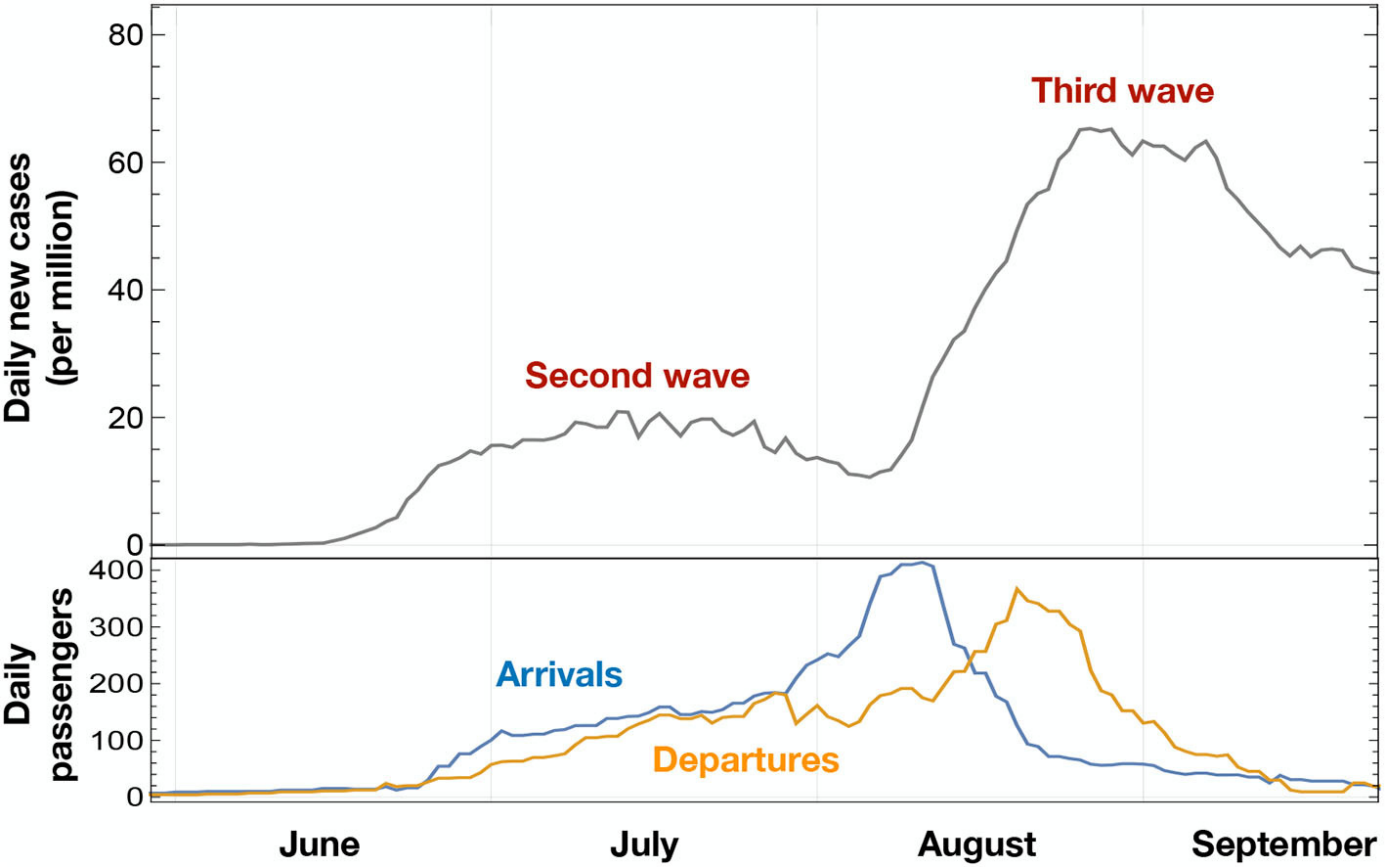
COVID-19 new cases (gray line) in Croatia between June and September, 2020, shown as weekly averaged data. As a comparison, we report the number of maritime passengers arriving or departing from Croatian ports in the same period.

As a case study, we considered Croatia, with a special focus on the impact of maritime transportation. It has been shown that maritime transportation has been crucially affected by the COVID-19 pandemic (27), with an impact also on its greenhouse gas emission (28). The initial cases in Croatia were reported in March-April 2020. Later on, both a second and third wave of infections occurred between June and September 2020. As shown in Figure 1, this coincided with the reopening of the sea-based touristic links. Hence, it is a natural question to ask if the reopening of maritime routes can be traced as the main cause of the restart of the infection exponential increase. Maritime passenger transportation is crucial for Croatia due to its long and archipelagic coast with more than one thousand islands on the Adriatic Sea. Regular ferry traffic between Croatia and Italy is extremely significant and it takes place via the ports of Split, Zadar, and Dubrovnik (29), connecting them with the Italian ports of Ancona and Bari. Many tourists reach the Croatian coasts by this means. For this reason, ferries may have played the role of a superspreader, triggering the pandemic wave that hit Europe in the summer of 2020. It is worth noting that the situation in Croatia in June-August of 2020 is of particular interest, as it showed an earlier increase of infections after the lockdowns were lifted, as compared to other European countries (15). We remark that passenger mobility plays a crucial role in the diffusion of infections in the early phases of a pandemic, while at later stages variants due to genetic mutations start becoming the predominant factor in the emergence of new epidemic waves (30). Knowing more about the scenario at stake during this wave could help improve future measures to reduce efficiently the seed of the spreading and avoid costly measures with lower impact.

To reach the main objective of this study, we performed a data-driven analysis that quantifies the impact on the diffusion of COVID-19 of both terrestrial and maritime passenger transportation between Croatia and its neighboring countries. The role, if any, of maritime transportation is not easily assessed ex-ante. On the one hand, the amount of passengers carried by sea was smaller than that of other modes of transportation. The data shows that maritime passengers have been <1% of the car passengers entering and leaving Croatia in 2020. On the other hand, ferry passengers were transported in limited volumes for relatively long times, which might have favored the diffusion of the virus (31). This is also confirmed by more general studies in closed rooms (22–25), as often the case on board. The latter hypothesis would also be suggested by the timing of the infection diffusion across summer 2020, coinciding with the reopening of ferry connections across the Adriatic.

The datasets used in this study included the passenger flow to Croatia via waterborne, airborne, and terrestrial transportation modes. To connect mobility to the epidemiological data, we employed a novel approach to infectious disease spreading, the epidemiological Renormalization Group framework (eRG) (32), which is inspired by theoretical high energy physics (33, 34). The eRG offers a computationally inexpensive characterization of a single wave diffusion in terms of just two constant parameters. Once extended to a network of semi-isolated populations (14), it will enable to study the spread of the infectious disease among regions/countries. This method was pivotal in predicting the 2020 second wave in Europe (15). The main advantage of the eRG approach is the ability to characterize a single wave in an isolated region in all its phases, from the initial exponential increase to the peak and reduction of the new infections, in terms of a simple logistic function. It is, however, not well-suited for short-term forecasting (35), for which more traditional compartmental models are preferable (36). It is noteworthy that the eRG solutions are related to the simplest compartmental model, based on Susceptible-Infectious-Removed (SIR), with time-dependent parameters (37). For our purpose, the eRG offers a reliable handle to quantify the impact of mobility on the timing of the peak of the third wave in different regions of Croatia.

Other methods were previously employed to study spreading of infection diseases through interaction between different geographical regions. It is worth mentioning the different methods among which lattice simulation using SIR models with space extension and interaction using Bayesian maximum entropy theory (38), lattice spatio-temporal modeling framework integrating SIR and log-Gaussian Cox process (LGCP) process (39), graph modelization where policies to curb the spreading are tested by removing individuals (40) or agent-based simulations of aerosol and pedestrian trails to track the spreading at the level of an airport (41). The eRG method is more economical as it only considers the total number of infections, and it has been used in conjunction with airborne traffic to study the early diffusion of COVID-19 in the United States (42).

The remainder of this paper will outline the methodology, in Section 2, and the results, in Section 3. Conclusions and recommendations will be described in Section 4.

## 2. Methodology

To study the diffusion of the COVID-19 infections in Croatia, we combined data describing people mobility via various transportation modes with an epidemiological dataset. The latter consists of the daily number of newly infected individuals that were tested positive, during a period of time, in each county (43) of Croatia. The mobility data comprises the number of individuals entering Croatia via sea, land or air, provided by various sources and described in detail in Table 1. The correlation between the two datasets was studied within the eRG framework, consisting of a set of coupled differential equations (14). The main advantage of the mathematical model provided by the eRG is to allow characterization of epidemiological waves in terms of a limited number of parameters. The model also includes the diffusion of an infectious disease within connected regions. Hence, the position of the peaks, i.e., the timing of the local maxima of new infections in different regions, can be predicted as a function of the mobility data. Comparing the predictions of the model with the actual data allows us to determine the role of various transportation modes in facilitating the diffusion of COVID-19. This mechanism is expected to be the dominant mechanism of diffusion at the beginning of the pandemic. In Croatia, the first epidemiological wave, characterized by a temporary exponential increase of the cases, took place in March through April 2020. After a period when the rate of infections slowed down due to the lockdowns, a new increase was detected starting toward the end of June and lasting through the end of July, followed by another increase in August and September. We identify these two episodes as the “second” and “third waves,” respectively (see Figure 1).

**Table 1:** Description of mobility and epidemiology datasets used in this work.

	**Vehicles**	**Name**	**Provider**	**Resolution**		**No. of datapoints**
				**Time**	**Space**	
Maritime	Ferries (Ro-Pax)	CIMIS	MMPI	Daily	By port	1,360
Terrestrial	Cars	Highway data	UNIRI	Summer	Borders	108
	Trains	Railway data	UNIRI	Annual	Borders	14
Airborne	Planes	Air traffic data	MMPI	Monthly	Airports	21
Epidemiology	−	New cases	MMPI	Daily	County	575

For each of the five datasets, the table summarizes the data provider (being the Ministry of maritime affairs, transport, and infrastructure of Croatia—MMPI—and the University of Rijeka—UNIRI) and the spatial/temporal resolution of the datapoints. In the case of summer and annual resolution, only one point per geographical unit is provided. For terrestrial data, information is provided for every entry point on the external borders of the country. The last column indicate the number of datapoints used in the study, comprising of both temporal and spatial units. More details are provided in Section 2.1.

The rest of this section provides a detailed presentation of the datasets used for this research in section 2.1, the procedure for a geographical aggregation of the data, outlined in section 2.2, and the application of the eRG framework to this specific data in section 2.3.

### 2.1. Datasets

The following datasets for 2020 (see summary in Table 1) were used for the numerical analyses:

(1) Maritime transportation data was obtained from the *Croatian Integrated Maritime Information System* (CIMIS). The dataset, provided by the GUTTA partner “*Ministarstvo Mora, Prometa i Infrastrukture*” (MMPI—Ministry of Maritime Affairs, Transport, and Infrastructure of Croatia), contains information for each Croatia seaport regarding departures and arrival times of ferries along with the number of both embarking and disembarking passengers. We included data from car-passenger ferry routes, along the routes Ancona-Zadar, Ancona-Split, and Bari-Dubrovnik.
(2) Car traffic data was collected by the *University of Rijeka* (UNIRI) by contacting the limited liability company “Hrvatske Ceste” (44), which has a function of management, construction and maintenance of state roads. This dataset provides the number of travelers crossing each Croatian border control checkpoint per year. Hence, we reconstructed the average flow between the neighboring countries (Slovenia, Hungary, Serbia, Bosnia-Herzegovina, Montenegro) and the considered Croatian regions, both entering and leaving the country by road. The data also contains the number of travelers passing various checkpoints along the major Croatian roads inside the country, however this information was discarded as it did not allow to reliably reconstruct people mobility within Croatia.
(3) Railway traffic data was extracted from the “*Independent Regulators' Group*” IRG-rail 2021 report (45), provided by MMPI and from *HŽ Infrastruktura*. The latter organization is responsible for the railway system in Croatia. The 2021 report also outlines the impact of the COVID-19 crisis on the network during the first half of 2020. Data of network topology, passenger flows, and operational conditions was taken from the report. The railway traffic was recorded on an annual basis.
(4) Air traffic data was procured by the MMPI from the *Eurocontrol Air Traffic Directorate* in Lyon, France. The dataset consisted of the number of travelers per month per airport in Croatia for 2020.
(5) The COVID-19 epidemiological data was extracted from an open-source Croatian public resource, “koronavirus.hr” (46). The dataset includes the cumulative total number of infections and the number of daily new infected individuals for each Croatian county. A new infection is counted for each individual that reported a new positive test. We extracted the data from the 21st of March, 2020, to the 18th of October, 2021, corresponding to 575 days in total. The raw data was pre-processed to smooth daily fluctuations by applying a moving 7-day averaging procedure.

**Figure 2:**
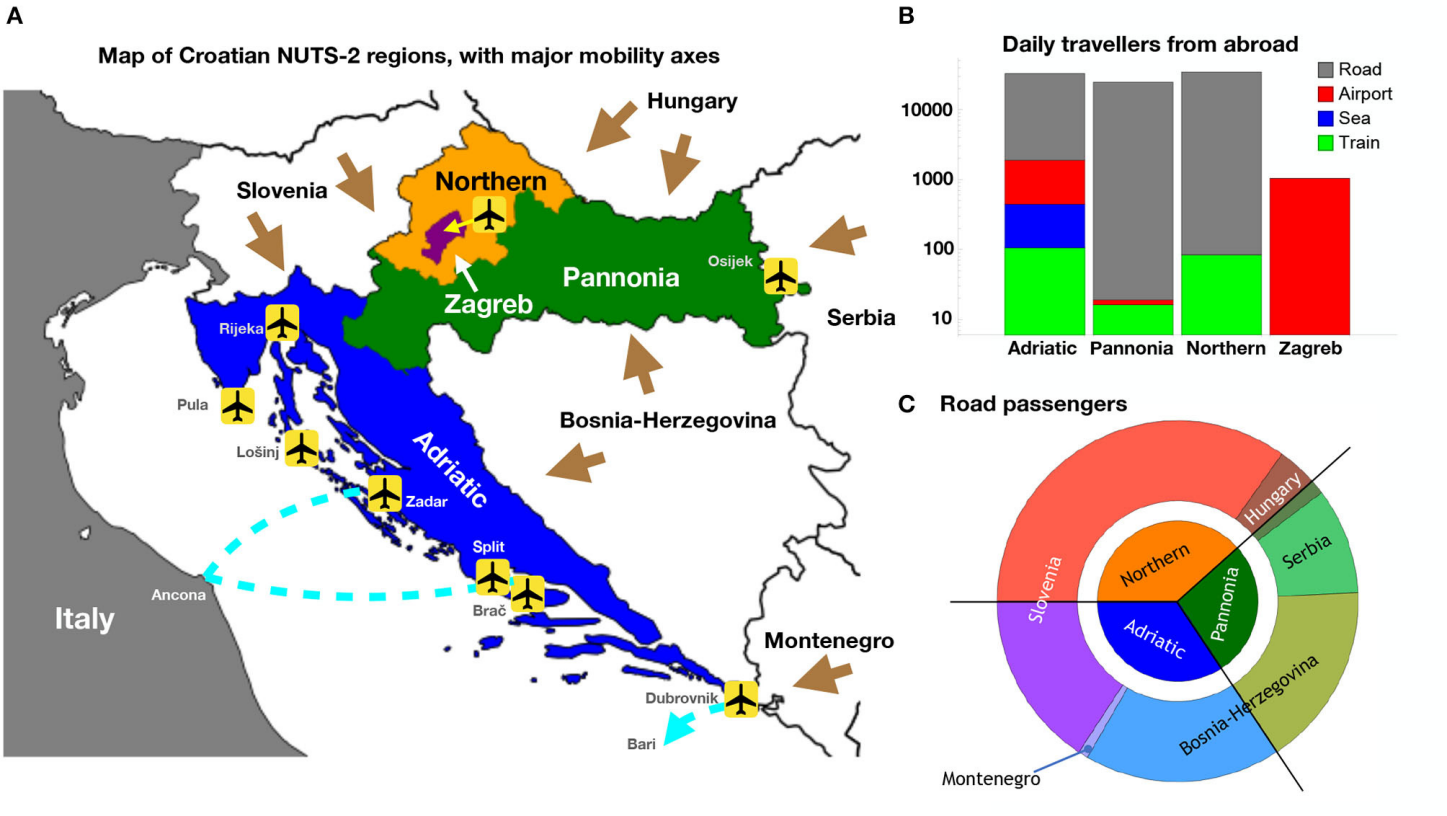
**(A)** NUTS-2 regions of Croatia, indicating the main mobility axes via land (brown arrows), flights (international airports at Zagreb, Osijek, Rijeka, Pula, Lošinj, Zadar, Split, Brač, and Dubrovnik) and sea (car-passenger ferry lines in cyan). **(B)** Partition of the passenger inflow from abroad to the four NUTS-2 regions. The histograms are stacked in log scale, showing a clear dominance of road passenger flow. **(C)** Partition of the road passenger flow among Croatian regions and the neighboring countries. The outer ring shows the flow from each country, proportionally distributed to the regions.

We offer a visualization of the mobility data in Figure 2, subdivided according to the regions of Croatia (“Pannonia,” “Adriatic,” “Northern,” “Zagreb”) we define in the next section. In particular, in Figure 2B we show the average number of daily passengers entering each region from abroad, plotted as stacked histograms in log scale. This proves that the dominant flow is due to cars, followed—one order of magnitude below—by the airborne traffic. The only exception is Zagreb, which is an enclosed region, hence its only direct connection with other countries is through airborne transport. The maritime passenger flow is only relevant for the Adriatic region, where it only constitutes roughly 1% of the total. Finally, Figure 2C shows a subdivision of the car passengers by region, as shown in the inner circle, and by neighboring country, as shown in the outer ring. Note that the angle of each wedge is proportional to the number of daily passengers. The inner circle visualizes the proportion of the total passengers entering Croatia from the five neighboring countries, divided among the three relevant Croatian regions (Pannonia, Northern, and Adriatic). The ring shows the proportion of such passengers from each bordering country, highlighting the proportional distribution of passengers from Slovenia to Northern and Adriatic (no significant flow to Pannonia is observed in the data), from Hungary to Northern and Pannonia, and finally from Bosnia-Herzegovina to Pannonia and Adriatic, with addition to Serbia leading only to Pannonia and Montenegro only to Adriatic.

### 2.2. NUTS-2 regions and how to deal with Zagreb

Croatia is a diverse country in terms of geography, population density, and demography. It consists of an elongated coastal region of great touristic interest, and an internal region where the capital city Zagreb is located. Furthermore, it borders with Slovenia and Hungary in the North, Serbia in the East, Bosnia-Herzegovina, and Montenegro in the South. It also shares a maritime border with Italy in the west (across the Adriatic Sea). The main connections with abroad, therefore, are realized via road and railway, via ferries (with Italy mainly) and flights.

To characterize the diffusion of the virus within Croatia, we need to first establish regions within Croatia to be associated to the eRG equations. One possibility is to consider the Croatian counties, taking into account the subtlety of the mobility within counties and with neighboring countries. This corresponds, in the Eurostat nomenclature (43), to the “NUTS-3” level. However, for our purposes, this subdivision level would be problematic for multiple reasons. Firstly, the population of different counties is highly unequal, ranging from 50,000 inhabitants in *Lika-Senj* to 800,000 in *Grad Zagreb*. This unequal distribution of the population would amplify, for counties with low population, any statistical fluctuations in the epidemiological data. Furthermore, the unequal weight of different counties can bias the numerical output of the eRG computations. Secondly, it is challenging to quantify the mobility flows between counties due to the small size of these territorial units. Access to the flow data along main highways was provided by the MMPI. However, this data would not be sufficient to provide a reliable estimate at county level. In fact, drivers passing through the checkpoints on the main roads may drive across several counties, where possible contacts with infected individuals could take place. As a consequence, we deemed this level of geographical granularity to be inappropriate for our purposes.

We instead opted for the 2021 NUTS-2 level, where Croatia is subdivided into four statistical regions as shown in Figure 2A:

- Pannonia (HR02, *Panonska Hrvatska*): 1, 054, 000 inhabitants, stretching to the East and adjoining Hungary, Serbia and Bosnia-Herzegovina.
- Adriatic (HR03, *Jadranska Hrvatska*): 1, 372, 000 inhabitants, comprising of the coastal region and bordering Slovenia, Bosnia-Herzegovina and Montenegro, including Italy via sea.
- Northern (HR06, *Sjeverna Hrvatska*): 813, 000 inhabitants, in the north and bordering Slovenia and Hungary.
- Zagreb (HR05, *Grad Zagreb*): 800, 000 inhabitants, enclosed within the Northern region.

This division is much more uniform in terms of population, hence minimizing the statistical uncertainties of the data. It is also more suitable for studying the diffusion of the infectious disease, as each region has its own specificity that makes its role unique. For instance, Adriatic is the only region that is connected by maritime transportation as it encompasses all the ports of Croatia. Furthermore, the main international airports are in Adriatic (Split) and Zagreb. All regions except Zagreb are connected to neighboring countries via border road and train connections.

The above information highlights an issue with the mobility data related to Zagreb: the absence of a region border with abroad. However, the traffic to the capital city is expected to be of major importance. To consider this missing information, in the numerical results, we “re-routed” part of the road and train flow across boundaries to the Zagreb region. By this it is meant that part of the passengers traveling via car (or train) from abroad to, e.g., Pannonia are supposed to end up their journey not in Pannonia but in the Zagreb region. Same goes for Northern and Adriatic. This is justified by the fact that highways and railway lines connect the boundaries directly to Zagreb, while crossing any of the other three NUTS-2 regions of Croatia. We will investigate a few scenarios where a fixed fraction of the road and railway traffic from the surrounding regions is attributed to Zagreb.

### 2.3. Applying the epidemic Renormalization Group framework

The eRG framework (32) was formulated as a simple mathematical tool to describe the exponential increase in the number of new infections, followed by a reduction back to approximately zero. We will refer to this phenomenon as a “wave.” As seen from Figure 1, Croatia experienced three waves of COVID-19 between March and September 2020. More waves followed, showing larger numbers of infected individuals. It should be remarked, however, that the absolute number of cases in each wave cannot be completely trusted, as it depends on both the number of people that are subjected to tests and on biases in the testing strategies (e.g., correlations with hospitalizations, presence of asymptomatic cases, etc.).

For a single isolated region, with constant population during the spread of a single wave, the eRG framework provides a first-order differential equation to describe the time-evolution of the number of individuals that contracted the disease. The eRG equation reads


(1) $$\begin{array}{l} \frac{d\alpha }{dt}=\gamma \;\alpha \left(1-\frac{\alpha }{A}\right), \end{array}$$

where *t* is time and α is a non-dimensional function of the cumulative number of infected individuals in the region, *I*_*c*_(*t*). Hence, α(*t*) is a function of time only, where the spatial dependence has been integrated in. In principle, α can be any monotonic function of *I*_*c*_, however comparison with data for COVID-19 and SARS showed that an optimal fit can be obtained for the natural logarithm (32)


(2) $$\begin{array}{l} \alpha \left(t\right)=\ln\frac{I_{c}\left(t\right)}{N_{m}}\equiv \ln I_{n}\left(t\right), \end{array}$$

where we normalized the number of infections by the population of the region in millions, *N*_*m*_. Hence, *I*_*n*_ measures the number of infections per million inhabitants. The eRG Equation (1) depends on two constant parameters, γ and *A*, which embed different characteristics of an epidemiological wave:

- The γ parameter is an effective infection rate, measured in units of *t*^−1^. It describes how quickly the infectious disease spreads within the population of the region, and it does not depend on the effective number of total infections. As such, γ values from different regions can be compared. The numerical value of γ encodes all the effects that influence the diffusion speed: the transmissibility of the virus, social, and behavioral effects (47), and pharmaceutical interventions like vaccinations (42). All these effects are captured by a single and constant value over the development of a specific wave.
- The *A* parameter corresponds to the value of α at the end of the wave, hence it is a measure of the normalized number of infections in the region at the end of the wave. In fact, *A* = α(∞) = ln *I*_*n*_(∞). The significance of this parameter is affected by biases in the data collection in each region: for instance, the testing rates and policies. However, as long as these biases remain approximately constant during the development of a wave, the eRG approach can be effectively applied.

Note that γ does not depend on the number of infected individuals. As such, it does not suffer from biases coming from the number of available test kits, nor from testing policies adopted during various phases of the pandemic, nor on any possible regional differences. Hence, γ offers a reliable characterization of the severity of each wave in different regions and at different times. An advantage of the method lies in the fact that just two parameters (γ, *A*) suffice to characterize the wave, and they remain practically constant over the evolution of a single wave (42, 47). The values of γ and *A* can be obtained by fitting the epidemiological data in a specific region with the solution of Equation 1, which is the following logistic function:


(3) $$\begin{array}{l} \alpha \left(t\right)=\frac{Ae^{\gamma \left(t-t_{0}\right)}}{1+e^{\gamma \left(t-t_{0}\right)}}, \end{array}$$

where *t*_0_ is an integration constant setting the overall timing of the wave.

In previous works, the eRG framework was extended to include mobility of people among different regions, as long as the flow only involves a small fraction of the region inhabitants (14). This extension allowed to study the relation between the emergence of epidemiological waves in different regions, along with the mobility of people among regions. In particular, the timing of the wave peaks could directly be related to the mobility flow, providing a handle to quantify the impact of various transportation modes on the COVID-19 diffusion.

This feature of the multi-region eRG equations allows us to quantify the impact of the various transportation modes on the diffusion of the infection in the regions of Croatia. The eRG now provides a set of coupled differential equations, one for each *i*-th region under consideration (14, 15):


(4) $$\begin{array}{l} \frac{d\alpha_{i}}{dt}=\gamma_{i}\;\alpha_{i}\left(1-\frac{\alpha_{i}}{A_{i}}\right)+\underset{j}{\sum }\frac{k_{ij}}{N_{m,i}}\left(e^{\alpha_{i}-\alpha_{j}}-1\right),\;\alpha_{k}=\ln\frac{I_{k}\left(t\right)}{N_{m,k}}, \end{array}$$

where *k*_*ij*_ represents the number of travelers per million inhabitants going from region *i* to region *j*. The second term in Equation 4 describes the change in the number of infections in the *i*-th region due to the travelers leaving and entering the region. It is assumed that the rate of infected individuals among the travelers is the same as in their region of origin, leading to the proportionality to the number of infected *I*_*k*_(*t*) in the two connected regions, expressed in Equation 4 in the exponential form. The above set of equations also permits the addition of a *source region*, i.e., a population of infected individuals that ignites the spread of the infections in the set of regions under consideration (15).

In the case of Croatia, the value of the parameters *k*_*ij*_ can be estimated by use of the mobility datasets in Table 1.

**Figure 3:**
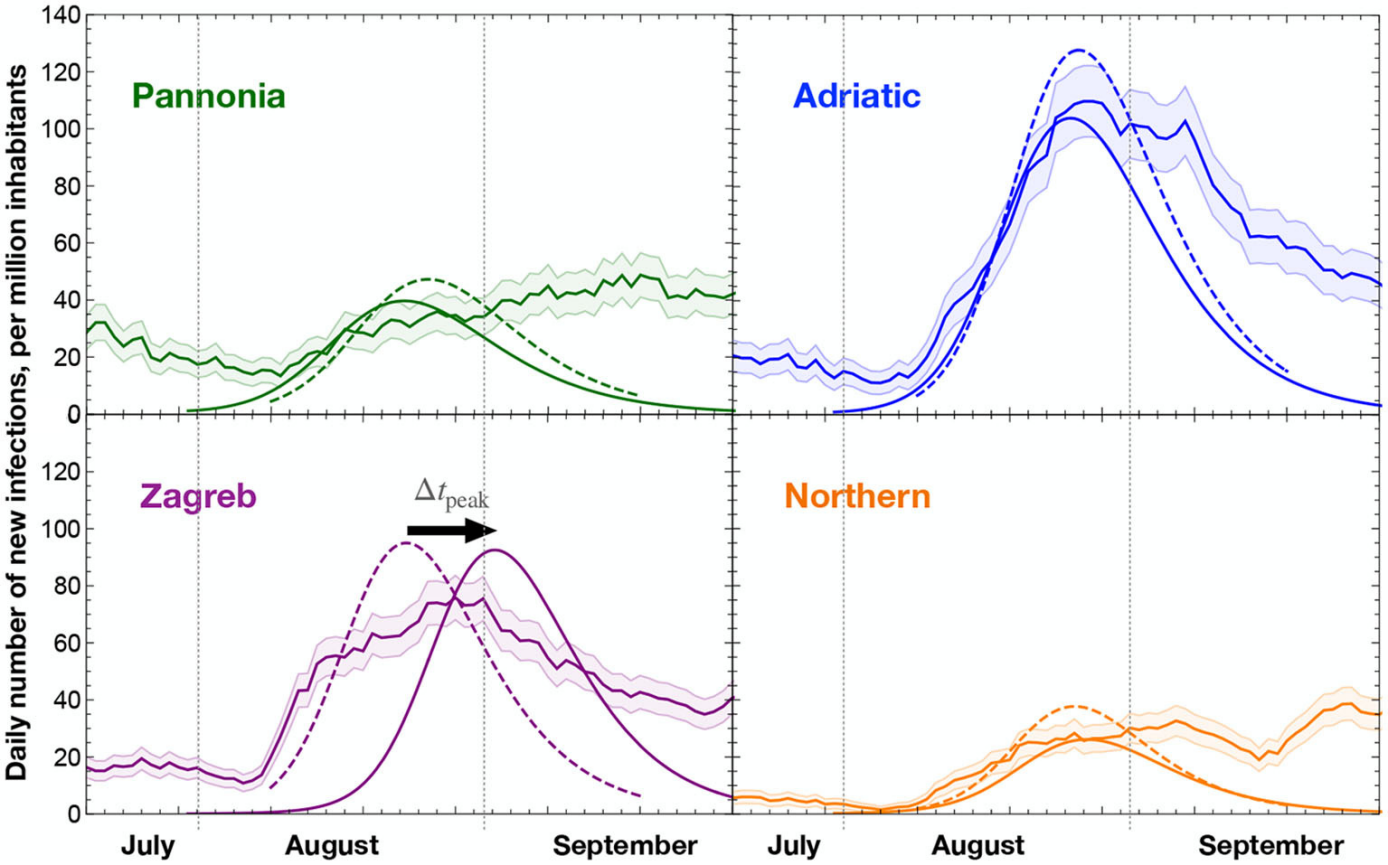
Comparison of the data (bands with statistical error), eRG modeling of the third wave (dashed curves), and the result of the eRG equations with mobility data (solid curves). The eRG computation includes all mobility data without relative weight in the number of passengers. The statistical error is computed following a Poissonian distribution on the number of daily new infections, giving an error of $\sigma =\sqrt{N}$ for a count of *N* new cases. The peak mismatch metric Δ*t*_peak_ is shown with its orientation for the case of the Zagreb region.

To quantify the impact of the mobility datasets listed in Table 1 on the diffusion of the third wave in Croatia, we adopted the following procedure:

- We subdivide Croatia into four regions, chosen to match the Eurostat 2021 NUTS-2 classification.
- For each wave, we fit the eRG parameters on the available epidemiological data in each region. For this study, we focus on the second and third waves, occurring between June and September 2020. The parameters γ_*i*_ and *A*_*i*_ are extracted from least-square fits and are listed in Table 2, where we also indicate the date where the peak occurred, as modeled by the eRG solution.
- We use the eRG equations, together with the fitted parameters, to numerically calculate the diffusion of the third wave to the different regions.
- As source regions, we use the neighboring countries (including Italy for the maritime transport) by coupling the eRG equations of the Croatian regions to their epidemiological data. For the flight transportation, we use the epidemiological data of the whole world as a source.
- We define and study specific scenarios where the different mobility datasets are included with a weight. The latter parameterizes the effective impact of the actual transportation modes on the virus diffusion. In practice, this weight corresponds to the probability of finding infected individuals among the passengers of that specific transportation vector.
- We compare the result of the numerical equations to the observed data, to establish which configuration offers the best fit to the timing of the third wave in the different regions. As an example, Figure 3 shows the third wave obtained by the eRG equations when all transportation passenger are included unweighted. As a quantifier of the model performance, we make use of the shift between the predicted wave peak and that of the epidemiological data, cf. Equation (5).

**Table 2:** Parameters *A* and γ (see Equation 3) extracted from the fits of the second and third wave in the four Croatian regions and used in the model in Equation (4).

	**Second wave**			**Third wave**		
**Region**	*A*	γ	*t*_peak_	*A*	γ	*t*_peak_
Pannonia	6.62	0.20	July 4	7.01	0.13	August 26
Adriatic	6.41	0.10	July 15	7.93	0.14	August 26
Zagreb	6.59	0.19	July 2	7.58	0.15	August 23
Northern	5.00	0.23	July 3	6.64	0.16	August 26

We also indicate the peak timing *t*_peak_ from the eRG modeling of the waves. They correspond to the peaks of the dashed lines in Figure 3. One can see that the third wave has a higher total number of cases for all regions at the end of the wave as can be easily seen in Figure 1, but smaller infection rate than the second wave. We also see that the four regions had more diverse infection rates during the first wave: this may be due to the different level of application of the mitigation measures imposed by the Croatian government in the early phases of the pandemic. Such differences became smaller for the third wave, when people were more accustomed with the measures.

Figure 3 shows that in some regions, like Pannonia and Northern regions, a peak of infections is not readily identified from the data. This is mainly due to the limited statistics available in these regions, while at national level (and in other regions of the world) it was apparent that the COVID-19 diffusion has a wave-like character. Hence, we identified peaks in the data by using the logistic function suggested by the eRG framework (Equation 3), and using the beginning of August as an initial time (*t* = 0). The resulting curves are shown in dashed lines in Figure 3, with the *t*_peak_ listed in Table 2. Then we compute the shift in the peak prediction from the mobility data as


(5) $$\begin{array}{l} \text{Δ}t_{\text{peak}}=t_{\text{peak}}{|}_{\text{eRG}}-t_{\text{peak}}, \end{array}$$

as indicated in Figure 3 for Zagreb. Hence, positive Δ*t*_peak_ indicates that the mobility data predicts a delayed peak as compared to the data, while an optimal modeling is achieved if Δ*t*_peak_ ~ 0. The numerical computations have been performed on a personal computer, using the Wolfram's software *Mathematica*.

## 3. Results

The results obtained via the eRG framework consider various configurations of the mobility data. In this way, two main research questions could be addressed: the role (if any) of cross border passenger flow via the maritime links and the estimation of the road traffic to and from Zagreb (Section 3.1). Both answers are established by studying the individual impact of each transportation mode. Finally, we study the combined effect of all mobility data and determine the optimal configuration to reproduce the epidemiological data (Section 3.2). In practice, given a set of mobility data, which feeds into the values of *k*_*ij*_ in the eRG equations, we compute the timing of the third wave in the four Croatian regions. To quantify the strength of the numerical solution, we compute the time difference between the peak in the solution and the peak observed in the data (obtained via the eRG modeling). We will see what configurations are best suited to represent the epidemiological data, and we will interpret the results in terms of the relevance of the various transportation modes. From previous work (14), we know that varying the weight of the passenger number can move the peak time by up to a week, hence empirically we consider an agreement to be good if the location of the peak (Δ*t*_peak_) is captured within 5 days.

### 3.1. Single transportation modes and role of maritime mode

**Figure 4:**
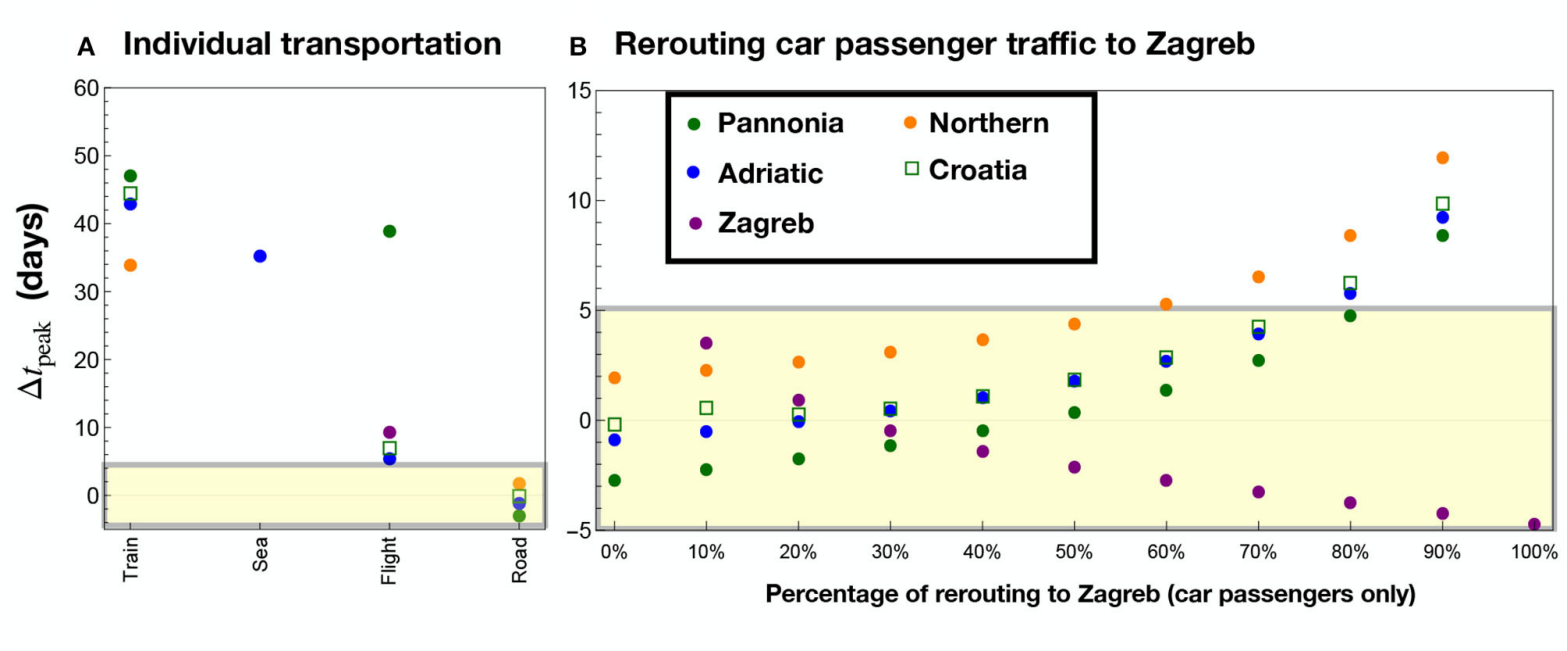
Results of the eRG computation when including only one transportation mode, with each column corresponding to a different combination of data. The region of acceptable peak mismatch (±5 days) is etched in yellow. In **(A)** one transportation mode is included: Train, Sea, Flight, and Road data. Missing points correspond to regions where no wave is ignited if just the transportation mode in parenthesis is considered: Pannonia (Sea), Zagreb (Train and Sea), Northern (Sea and Flights). Flights only marginally reproduce Zagreb and Adriatic, where the main international airports are located, while road transportation (without rerouting) fully misses Zagreb. In **(B)**, road data only is considered, with the indicated percentage of passengers rerouted from all other regions to Zagreb.

Our aim is to establish what are the main effects of each type of mobility on the infection diffusion. To achieve this, we solved the eRG equations with a single transportation type (“Train,” “Sea,” “Flight,” “Road”) to understand how the data can be reproduced by only using them one at time. To quantify the fitness of the eRG calculation, in Figure 4 we show the peak time differences, in days, in each of the four regions, indicated by the solid dots. Figure 4A refers to results obtained including a single dataset, where the missing points indicate regions where a wave start was not triggered by use of just a single specific transport mode.

The results show that none of the transportation modes alone can reproduce the data. In particular, “Road” data fails to ignite the wave in Zagreb due to the lack of borders with abroad. Flights play an important role for both Adriatic and Zagreb, where the major international airports are located, while maritime and train transport do not play any significant role in the diffusion of infections. It is important to highlight that we also report with a square symbol the cumulative data for the whole of Croatia. This was obtained by solving the eRG equations for Croatia as a single region. These results clearly indicate that both railway and maritime passengers had a negligible impact on the diffusion of COVID-19 in Croatia.

As a second step, we tested the effect of rerouting a fixed percentage of the road data from the other regions (Adriatic, Pannonia, and Northern) to Zagreb. While Adriatic is quite far from Zagreb, we consider that a major road connection to Slovenia (and Italy) goes through the border of the Adriatic region, connecting Zagreb with Rijeka.

The results are shown in Figure 4B, and are labeled by the percentage of rerouting. It is seen that a rerouting level between 10 and 40% can well reproduce the epidemiological data for all regions of Croatia (|Δ*t*_peak_|<4 days). This shows that the diffusion of the virus during the third wave in Croatia can be well-modeled by using road-only data. In the next section we analyse a more realistic scenario where all transportation modes are included, with the main goal of validating the main conclusions of this analysis.

### 3.2. Combined analysis and optimal traffic diversion onto Zagreb

**Figure 5:**
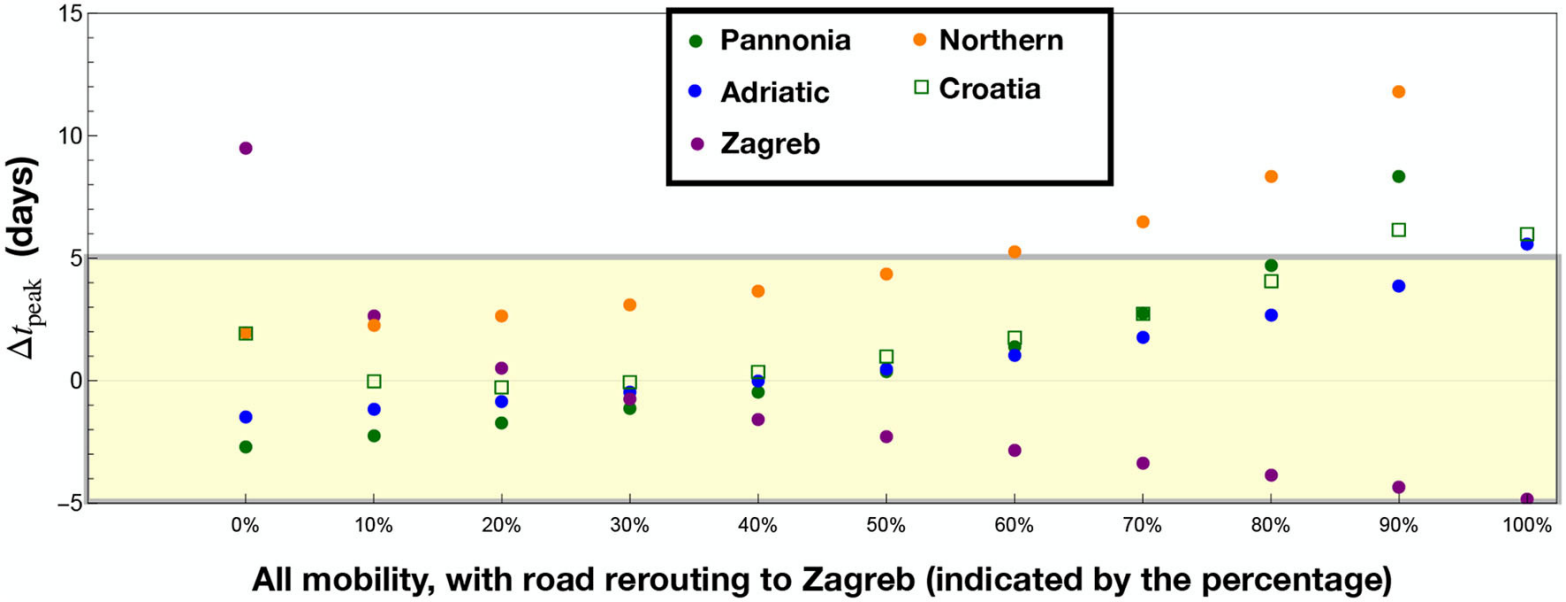
Results including all transportation modes, with a given rerouting from Adriatic, Northern, and Pannonia to Zagreb of the road passengers. The inclusion of all transportation passengers improves the results for Pannonia and Adriatic as compared to Figure 4.

Having investigated the possibility of having a dominant mobility mode for the infection in Croatia, we can clearly see that road traffic is the main factor of virus propagation. As argued in Section 2.2, we had to assume that a percentage of the car traffic going from abroad to Croatia was directly rerouted to Zagreb. We simulated, therefore, a scenario where all mobility data are included, while a certain percentage of the road traffic is rerouted to Zagreb from the Adriatic, Pannonia, and Northern regions. This includes the impact of air traffic which can be relevant for both the Adriatic and Zagreb regions. The results are shown in Figure 5. It is still found that a rerouting in the range of 10–40% can optimally reproduce the epidemiological data in all regions of Croatia, but with smaller mismatches with respect to the results shown in Figure 4B.

We observe a marginal improvement for Pannonia and Adriatic compared to the road-only case, mainly due to the impact of airborne traffic. Considering Pannonia, Adriatic, and Zagreb, the best scenario is based on a 30% road traffic diversion to Zagreb, where we observe |Δ*t*_peak_| <2 days for those regions. Note that the Northern region is systematically delayed compared to the peak in the data: this could be due to airborne passengers landing in Zagreb but eventually directed to the Northern region. Another reason may be the poor modeling of the epidemiological data, where a clear peak is not well-visible for this region, as shown in Figure 3.

## 4. Conclusions

We have quantitatively analyzed mobility data along with epidemiological data during the period of June–September of 2020 in Croatia. The eRG framework was used to model the epidemiological data and numerically correlate them to the mobility data. The main goal was to establish the impact of various mobility vectors on the diffusion of the COVID-19 infections, at the origin of the third wave in Croatia between August and September 2020.

Our results show that, although the timing coincided with the restart of the maritime traffic from Italy after the first lockdown, maritime or train transportation did not play any significant role in the onset of the third wave in August 2020. Instead, we demonstrated that road mobility was the main contributor, as the car passenger fluxes, when integrated in the eRG framework, successfully reproduce the timing of the waves in all NUTS-2 regions of Croatia. However, to optimally reproduce the epidemiological data, we assumed that a fraction of the cross-border road passengers were directed to Zagreb, a region which does not have direct borders with neighboring countries. The inclusion of airborne passengers yields to optimally matching the data for the Pannonia, Adriatic, and Zagreb regions. This means that epidemic wave peaks are reproduced within an error of 3 days when about 30% of the car passengers are redirected to Zagreb. The Northern region of Croatia always features a delay in the eRG prediction, limited within the acceptable range of 5 days.

These results provide a further validation of the eRG method to combine mobility data with a fast and accurate prediction of the next epidemiological wave. However, due to lack of data, internal mobility within Croatia was not considered. This gap also relates to the cross-border passengers directed to Zagreb while crossing the other regions. Hence, the eRG results could be greatly improved if aggregated internal mobility data were provided, for instance based on smartphone usage and tracking (47).

Nevertheless, our study allowed us to deduce a couple of important lessons on the effect of various transportation modes for the diffusion of an infectious disease. First and foremost, the study warns about simplistic association of nearly simultaneous signals during a pandemic. Differently from what could be naively expected, no causal link between the ferry traffic and the onset of the third wave of COVID-19 in Croatia could be assessed. Instead, and this is our second finding, road traffic was found, during those early phases of the pandemic, to be the leading driver of the virus diffusion in Croatia. This points toward land border control as one of the most effective ways to limit the spread of an infectious disease in its early stages. However, this approach can be effective only if timely implemented (14). The eRG-based modeling approach in combination with proper mobility datasets can thus provide the means to rule out non-causal relationships, supporting decision-makers in recognizing the most effective actions at the beginning of a pandemic.

## Data availability statement

The raw data supporting the conclusions of this article will be made available by the authors, without undue reservation.

## Author contributions

DA and AJ collected and analyzed mobility data relative to train and car passengers. GC and CC designed the model and the computational framework, while CC provided the numerical results and data analysis. CC, GC, and GM led the writing of the manuscript. All authors participated in the design of this project. All authors contributed to the article and approved the submitted version.
